# Functional expression of a novel Kunitz type protease inhibitor from the human blood fluke *Schistosoma mansoni*

**DOI:** 10.1186/s13071-015-1022-z

**Published:** 2015-08-04

**Authors:** Shiwanthi L. Ranasinghe, Katja Fischer, Geoffrey N. Gobert, Donald P. McManus

**Affiliations:** Molecular Parasitology Laboratory, QIMR Berghofer Medical Research Institute, Brisbane, QLD Australia; School of Public Health, The University of Queensland, Brisbane, QLD Australia

**Keywords:** Kunitz type protease inhibitor, SmKI-1, *Schistosoma mansoni*, Anti-coagulant

## Abstract

**Background:**

Schistosomes are able to survive for prolonged periods in the blood system, despite continuous contact with coagulatory factors and mediators of the host immune system. Protease inhibitors likely play a critical role in host immune modulation thereby promoting parasite survival in this extremely hostile environment. Even though Kunitz type serine protease inhibitors have been shown to play important physiological functions in a range of organisms these proteins are less well characterised in parasitic helminths.

**Methods:**

We have cloned one gene sequence from *S. mansoni*, Smp_147730 (*SmKI-1*) which is coded for single domain Kunitz type protease inhibitor, *E. coli*-expressed and purified. Immunolocalisation and western blotting was carried out using affinity purified polyclonal anti-SmKI-1 murine antibodies to determine SmKI-1 expression in the parasite. Protease inhibitor assays and coagulation assays were performed to evaluate the functional roles of SmKI-1.

**Results:**

SmKI-1 is localised in the tegument of adult worms and the sub-shell region of eggs. Furthermore, this Kunitz protein is secreted into the host in the ES products of the adult worm. Recombinant SmKI-1 inhibited mammalian trypsin, chymotrypsin, neutrophil elastase, FXa and plasma kallikrein with IC_50_ values of 35 nM, 61 nM, 56 nM, 142 nM and 112 nM, respectively. However, no inhibition was detected for pancreatic elastase or cathepsin G. SmKI-1 (4 μM) delayed blood clot formation, reflected in an approximately three fold increase in activated partial thromboplastin time and prothrombin time.

**Conclusions:**

We have functionally characterised the first Kunitz type protease inhibitor (SmKI-1) from *S. mansoni* and show that it has anti-inflammatory and anti-coagulant properties. SmKI-1 is one of a number of putative Kunitz proteins in schistosomes that have presumably evolved as an adaptation to protect these parasites from the defence mechanisms of their mammalian hosts. As such they may represent novel vaccine candidates and/or drug targets for schistosomiasis control.

**Electronic supplementary material:**

The online version of this article (doi:10.1186/s13071-015-1022-z) contains supplementary material, which is available to authorized users.

## Background

Despite constant contact with immune components in the human blood system, adult schistosome worms are capable of surviving for prolonged periods in the mesenteric veins of humans, in some instances, more than 30 years, without triggering host inflammatory reactions or promoting thrombus (blood clot) formation [1]. It has been demonstrated that a whole worm extract of *S. mansoni* prolonged activated partial thromboplastin time (APTT) and was able to specifically block the enzymatic activation of factor XI (plasma thromboplastin antecedent; PTA) by factor Xlla (activated Hageman factor) [2] but the precise molecule(s) involved have not been identified. It is, however, likely that schistosome protease inhibitors play a role in interacting with the proteases involved in blood coagulation. In general, parasitic helminths cause chronic disease and have evolved remarkable capabilities to down regulate host immunity, thereby ensuring their survival within their mammalian hosts [3]. Activated mammalian leukocytes produce several substances including cationic proteins, hydrolytic enzymes (mainly elastase and cathepsin G) and oxidants all of which can cause damage to schistosomes. It is recognised that schistosomula, lung-stage schistosomula, and adult worms are sensitive to both pancreatic elastase and neutrophil elastase [4]. In turn, to counteract the effects of elastase, schistosomes produce protease inhibitors, the best example so far known being a serpin, *Smpi56* [5]. Kunitz type protease inhibitors also possess the ability to inhibit several proteases, so their functional characterisation is also important. On a practical level, it has been suggested that studies on protease inhibitors can advance the understanding of host-parasite biology and lead to the identification of novel vaccine candidates and/or drug targets against schistosomes [6].

The Kunitz type protease inhibitors are ubiquitous in almost all eukaryotes [7–12], with bovine pancreatic trypsin inhibitor (BPTI) being the first described [13]. These proteins possess one or more Kunitz domains, with the Kunitz-type motif consisting of approximately 60 amino acids, and having six conserved cysteine residues which connect in a characteristic disulphide bonding pattern (C1-C6, C2-C4, and C3-C5) [14]. The amino acid residue at the P_1_ reactive site [15] is the major determinant of the energetic and specificity of protease recognition by Kunitz inhibitors; typical trypsin inhibitors contain Arg (R) or Lys (K) at the P_1_ site whereas typical chymotrypsin inhibitors contain Leu (L) or Met (M) [16]. In invertebrates, Kunitz inhibitors have been shown to be involved in a range of physiological processes including blood coagulation, fibrinolysis, inflammation and ion channel blocking [17]. However, there is limited information on the Kunitz inhibitors of parasitic helminths in general [8, 18–20], and none on schistosomes, so that functional characterisation of *S. mansoni* Kunitz proteins may shed light on their role in the host-parasite interplay.

We identified several gene sequences encoding Kunitz type protease inhibitors by interrogation of the available genome sequence data for *S. mansoni*. Among these, one gene (Smp_147730), having been previously shown to be highly up-regulated in schistosomula by RNA-seq transcriptome profiling [21] was selected and expressed in *E. coli* and purified. This Kunitz inhibitor, designated SmKI-1, is the first to be functionally characterised from *S. mansoni*; it inhibits trypsin, chymotrypsin, neutrophil elastase, FXa and plasma kallikrein, and prolongs both APTT and prothrombin time (PT) for blood clot formation, suggesting possible roles in both anti-inflammatory and anti-coagulation processes.

## Methods

### Ethics statement

All animal experimentation was conducted in strict accordance with protocols approved by the QIMR Berghofer Medical Research Institute (QIMRB) Animal Ethics Committee (project number P242), which adheres to the Australian code of practice for the care and use of animals for scientific purposes, as well as the Queensland Animal Care and Protection Act 2001; Queensland Animal Care and Protection Regulation 2002.

### Parasite materials

The Puerto Rican strain of *S. mansoni* is maintained in Animal Resource Centre (ARC) Swiss mice and *Biomphalaria glabrata* snails at the QIMRB animal facility from stocks provided by the Schistosomiasis Resource Centre, Biomedical Research Institute, Rockville, Maryland, USA. *Biomphalaria glabrata* snails were induced to shed by exposure to bright light and cercariae were concentrated by incubating the tubes in which they were contained on ice for 0.5–1 h. Schistosomula were mechanically transformed from cercariae using a standard protocol [22]. Adult worms were perfused from ARC Swiss mice using sodium citrate buffer (0.15 M sodium chloride, 0.05 M tri-sodium citrate) 7 weeks after cercarial challenge and washed three times with perfusion buffer to remove excess blood products. *S. mansoni* eggs and miracidia were isolated from infected mouse livers as described [23]. In brief, collagenase B is used to degrade the interstitial matrix of mouse liver tissue, after which the schistosome eggs are separated from the liver cells by 2 single-step density centrifugations through Percoll. Soluble parasite antigens were prepared by homogenising adult worms, cercariae, schistosomula, eggs and miracidia in PBS containing 1 mM EDTA and 1 mM PMSF on ice followed by centrifuging the homogenates at 16,000 g for 30 min at 4 °C. ES products from adult worms were obtained following the culture of *S. mansoni* worm pairs in perfusion buffer for 1 h at room temperature and subsequently collecting the supernatants [24].

### Cloning and expression of SmKI-1

Scrutiny of the *S. mansoni* genome, available at schistodb (http://schistodb.net/schisto/), resulted in the identification of several putative Kunitz type serine protease inhibitor gene sequences. Among these, one gene, Smp_147730 (*SmKI-1*), had previously been shown to be highly up-regulated in the transcriptomic profile of mechanically transformed schistosomula [21] and was thus selected for further characterisation. The amino acid sequence for SmKI-1 was validated by confirming the presence of start and stop codons, and SignalP 4.1 server [25] (http://www.cbs.dtu.dk/services/SignalP/) was used to check for the presence of a signal sequence. N-glycosylation site prediction was carried out with NetNGlyc 1.0 Server (http://www.cbs.dtu.dk/services/NetNGlyc/). Molecular weight and isoelectric point calculations were performed using the ExPASy-Compute pI/Mw tool (http://web.expasy.org/compute_pi/). The characteristic Kunitz protein domain was identified by searching the PROSITE database (http://prosite.expasy.org/) [26] and a multiple sequence alignment of SmKI-1 with other putative schistosome Kunitz proteins was generated with the Clustal Omega program (http://www.ebi.ac.uk/Tools/msa/clustalo/) [27]. Searches for similar protein sequences were performed using BLAST (http://blast.ncbi.nlm.nih.gov/Blast) on the NCBI (National Centre for Biotechnology Information) web site. Phylogenetic analysis [28] was undertaken using the putative *S. mansoni* Kunitz proteins and several other functionally characterised Kunitz proteins (http://phylogeny.lirmm.fr/phylo_cgi/index.cgi).

Primers with an introduced N-terminal 6 × His tag were designed and produced by Sigma® Aldrich (forward primer- 5ʹcatgccatggcacatcatcatcatcatcacgttagagacttgcattactcattgaatc3ʹ and reverse primer- 5ʹgatcctcgagctacacattgattctcattttacacactg3ʹ). The *SmKI-1* gene sequence was amplified using cDNA from adult worms and MyTaq™ DNA polymerase. Purified PCR products were digested with the restriction enzymes *Nco*I and *EcoR*I and ligated into the pET28a expression vector. The plasmid was transformed into *E. coli* BL21 (DE3) cells and a positive recombinant clone was grown in 5 ml of LB (Luria-Bertani) medium containing 30 mg/ml Kanamycin as the starter culture. Recombinant protein production was induced with 1 mM isopropyl β-D-1-thiogalactopyranoside (IPTG) at mid-log phase (A_600_ ~ 0.5-0.6) at 37 °C and samples were collected after 4 h post-induction. Harvested induced cells were lysed with lysozyme (10 mg/ml) in Tris buffer (100 mM NaH_2_PO_4_, 10 mM Tris-Cl) and homogenised using a Potter-Elvehjem homogeniser followed by sonication. Inclusion bodies were collected by centrifugation at 12,000 g for 20 min and then washed three times with Tris buffer containing 0.5 % (v/v) Triton-X 100. Inclusion bodies, solubilised in 6 M GuHCl, were allowed to bind to Ni charged resin (Novagen, Madison, WI, USA) at 4 °C. The flow through was collected and then the Ni column was washed sequentially three times with 4 ml Tris buffer containing 40 mM, 50 mM and 70 mM imidazole. Then, 50 ml of elution buffer (50 mM NaH_2_PO_4_, 300 mM NaCl), without imidazole, were allowed to pass through the column and, finally, refolded protein was eluted with the elution buffer containing 250 mM imidazole. The recombinant (r) SmKI-1 protein was electrophoresed on 15 % (w/v) sodium dodecyl sulphate polyacrylamide gels and its protein concentration determined using the Bradford assay [29].

### Real time PCR

Preparations of cDNA from adult male and female worms, eggs, miracidia, cercariae and newly transformed schistosomula were used for real time PCR. Real time PCR was performed using SYBR Green master mix (Applied Biosystems) on a Corbett Rotor Gene 6000 thermal cycler (Corbett Life Sciences). DNA segregation ATPase (TC15682 - Smp176580, Contig809759.1) [30] was used as the housekeeping gene for normalisation of the data. Each cDNA sample (25 ng per reaction) was tested in quadruplicate using forward (5ʹ tggtgaggaaactcggagac 3ʹ) and reverse (5ʹ cttccaaaatggccgtga 3ʹ) primers. All real time PCR reactions were carried out in duplicate, the confidence threshold (CT) of the second set of the results being normalised to the first set before evaluation. This was done by importing the standard curve of the first set to the second using Rotor-Gene 6000 software.

### Western blotting and immunolocalisation

Antigen affinity purified polyclonal antibodies against rSmKI-1 were custom made in mice by GenScript (Piscataway, NJ, USA). To test the specificity of the anti-rSmKI-1 antibodies, western blotting was first carried out with SmKI-1, BPTI (Roche diagnostics, Mannheim, Germany), *E. coli* expressed *Echinococcus granulosus* Kunitz protein (EgKI) (GenBank: EUB57880.1) and ulinastatin (human urinary trypsin inhibitor) which is composed of two Kunitz-type domains and functions as an anti-inflammatory compound (Prospec-Tany TechnoGene Ltd, Ness Ziona, Israel). rSmKI-1 and the other Kunitz proteins were separated on a 15 % (w/v) SDS-PAGE gel and transferred to an Immun-Blot® low fluorescence-PVDF membrane. Overnight blocking was performed with Odyssey buffer at 4 °C. Then, the membrane was subjected to incubation with the mouse anti-SmKI-1 anti-serum (1:2,000 dilution in Odyssey buffer and 0.1 % Tween-20) for one hour followed by incubation with IRDye-labeled 680LT goat anti-mouse antibody (1:15,000 diluted in Odyssey buffer with 0.1 % Tween-20 and 0.01 % SDS) for one hour on a shaker in a dark chamber. After a final wash with distilled water, the membrane was allowed to dry in the dark and visualised using the Odyssey® CLx Infrared Imaging System. Western blotting was also carried out using *S. mansoni* ES products and soluble adult antigens with the specific anti-SmKI-1 antibodies.

Tissue sections (4 μm) were adhered onto charged adhesive microscope slides from paraffin blocks of adult worms and mouse liver sections with trapped eggs. Following de-paraffinisation and rehydration, antigen retrieval was done with RevealtA solution (Biocare Medical, Concord, CA, USA). Then the tissue sections were blocked with 1 % (v/v) bovine serum albumin in TBS for 1 h at RT in a humidified chamber and incubated with anti-SmKI-1 antibodies (1:200) at 4 °C overnight. After three washes with TBS-T, the sections were incubated with Alexa fluor® 488 donkey anti-mouse IgG (1:500) (Invitrogen, Carlsbad, CA, USA) at 37 °C for 1 h. Nuclei in the tissue sections were counterstained with DAPIgold® (Invitrogen, Carlsbad, CA, USA) and observed under an EVOS® fluorescence microscope. Whole parasite mounts were also performed to check SmKI-1 protein expression in cercariae, schistosomula and miracidia, as described [31]. Briefly, the *S. mansoni* samples were fixed in 1.5 ml tubes with 4 % (v/v) phosphate buffered formalin for 25–30 min and then pelleted by a brief (~10-15 s) spin in an Eppendorf 5810R centrifuge (Hamburg, Germany) with brake settings on 0 or 1. Fixed samples were then permeabilised by rinsing three times with PBSTx (PBS with 0.3 % Triton X-100) and incubated in blocking solution (1 % BSA and 0.05 % Tween-20 in PBSTx) for 1.5-2 h. The subsequent steps were carried out as described for the paraffin block sections.

### Anti-coagulation and protease inhibition activity of SmKI-1

To test whether or not the blood coagulation pathways were functional, in the presence of rSmKI-1, three standardised tests, the activated partial thromboplastin time (APTT), the prothrombin time (PT) and the thrombin clotting time (TCT), were performed. These tests are widely used to determine anti-coagulation property of human blood [32]. APTT determines the deficiencies of intrinsic and common blood coagulation pathways, PT of extrinsic and common pathways, whereas TCT detects fibrinogen related disorders [32]. Plasma was separated from fresh human blood collected into sodium citrate vacutainers and incubated with different concentrations of SmKI-1 for 10 min in a 37 °C water bath. After adding CaCl_2_ to the mixture, the time taken for clot formation was measured by a Sta-R coagulometer (Diagnostica stago, Asnières sur Seine Cedex, France). TriniCLOT^TM^ APTT HS (Trinity Biotech, Bray, Co.Wicklow, Ireland), Thromborel®S (Siemens, Munich, Bavaria, Germany) and STA®-Thrombin (Diagnostica stago, Asnières sur Seine Cedex, France) kits were used for the determination of the APTT, PT [33–35] and TCT [36], respectively. Aprotinin and FVII negative plasma (Diagnostic Grifols, S.A., Spain) were used as the positive controls for APTT, TCT and PT, respectively. All tests were performed on two separate occasions each time using duplicate samples.

The protease inhibitor activity of rSmKI-1 was tested using a range of commercially available mammalian proteases: bovine trypsin, bovine chymotrypsin, porcine pancreatic elastase (PPE), human neutrophil elastase (HNE), human cathepsin G, activated coagulation factor X (FXa) and plasma kallikrein (PK). Each protease was incubated in the presence or absence of the rSmKI-1 protein in 96 well plates for 10 min. Then a chromogenic or fluorogenic substrate was added at concentrations ranging from 100 mM to 5 μM and the product formed was measured using a POLARstar OPTIMA microplate reader (BMG Lab Tech, Mornington, VIC, Australia) every min for 30 min.

Bovine pancreatic trypsin, bovine pancreatic α-chymotrypsin and the fluorogenic substrates *N*_α_-Benzoyl-L-arginine-7-amido-4-methylcoumarin hydrochloride and N-Succinyl-Ala-Ala-Pro-Phe-7-amido-4-methylcoumarin were purchased from Sigma Aldrich (St Louis, MO, USA). Trypsin and chymotrypsin assays were performed in 200 mM Tris–HCl (pH 8.2) containing 20 mM CaCl_2_ and 0.1 % (w/v) PEG 8000 at 37 °C. The kinetic rate of the substrate hydrolysis was measured at excitation/emission wavelengths of 370/460 nm. The activity of PPE was observed using the *Enzcheck*® elastase assay kit (Life technologies, Carlsbad, CA, USA) following the manufacturer’s instructions. Fluorescence signals were measured at 505/515 nm. HNE (SE284-0100) and Cathepsin G (SE283-0100), with the corresponding substrates N-Methoxysuccinyl-Ala-Ala-Pro-Val-7-amino-4-methylcoumarin (P224-0005) and Suc-Ala-Ala-Pro-Phe-pNA (P141-0025) respectively, were purchased from Enzolifesciences (NY, USA). The HNE assay was carried out with a buffer containing 100 mM HEPES, 300 mM NaCl and 0.05 % (v/v) Tween-20 (pH 8) at 25 °C with 2.5 nM enzyme and fluorescence signals were detected at 370/460 nm. Cathepsin G activity was measured in 100 mM Tris–HCl, 1.6 M NaCl buffer (pH 7.5) with 100 nM enzyme at 25 °C and the release of Pro-Phe-pNA was measured at 405 nm.

An activated Factor X inhibitor assay kit (Biovision Inc., Milpitas, CA, USA) was used to determine the inhibitory effect of rSmKI-1 on FXa and the fluorescence signals were detected at 350/450 nm. Human plasma kallikrein and fluorogenic kallikrein substrates (EMD Millipore, Billerica, MA, USA) were used to test for kallikrein activity in the presence or absence of rSmKI-1. Substrate hydrolysis was determined at 400/505 nm.

The values were corrected after subtracting background signals and all experiments were performed in triplicate.

Results were expressed as a percentage of the relative inhibitory activity of the rSmKI-1 protein using the formula:

$$ \mathrm{Percentage}\ \mathrm{of}\ \mathrm{relative}\ \mathrm{activity} = \left(\varDelta \mathrm{R}\mathrm{F}\mathrm{U}\ \mathrm{of}\ \mathrm{rSmKI}-1/\ \varDelta \mathrm{R}\mathrm{F}\mathrm{U}\ \mathrm{of}\ \mathrm{enzyme}\ \mathrm{control}\right)\ \mathrm{x}\ 100\% $$

∆ Relative unit (RU) = R_2_ - R_1_, Readings R_1_ and R_2_ were taken at t_1_ and t_2_ time points respectively, when the reaction was in the linear range. The half maximal inhibitory concentration (IC_50_) values were calculated using nonlinear regression with GraphPad Prism version 6.02 software.

## Results

### Analyses of SmKI-1

**Fig. 1:**
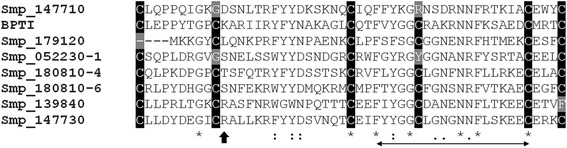
Clustal alignment of the Kunitz domains of Smp_147710, Smp_179120, Smp_139840, Smp_147730 (SmKI-1), the 1^st^ Kunitz domain of Smp_052230, the 4^th^ and 6^th^ Kunitz domains of Smp_180810 and BPTI. The six conserved cysteine residues are highlighted in black, the P_1_ site is shown by the black arrow head and the Kunitz family signature is shown as the double headed arrow

Sequence searches revealed the presence of seven putative Kunitz proteins in *S. mansoni* (Additional file 1). One contained two (Smp_052230) Kunitz domains while the remaining Kunitz molecules were associated with other domains such as chitin binding type-2 (Smp_180810) or spondin (Smp_180240), with Smp_180240 having two distorted Kunitz domains. The Kunitz domain of Smp_147710 did not contain the characteristic inhibitory amino acid at the P_1_ site and probably has an alternative function to protease inhibition. Two amino acid sequences with a single Kunitz domain (Smp_179120 and Smp_139840) were each devoid of two essential cysteine residues. Smp_147730 (SmKI-1 - Gene bank accession number CCD77156) was selected for further characterisation as it had a single secretory type Kunitz protein domain containing the inhibitory amino acid at the P_1_ site and was of full length. Furthermore, the Smp_147730 gene had been shown previously to be highly expressed in mechanically transformed *S. mansoni* schistosomula [21]. Clustal alignment (Fig. 1) of the Kunitz domains of these putative *S. mansoni* Kunitz proteins and BPTI showed that the amino acid sequence is highly conserved within the domain. Smp_180240 was excluded due to its two highly distorted Kunitz domains.

**Fig. 2:**
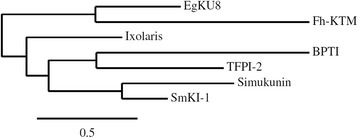
Phylogenetic analysis of SmKI-1 and functionally characterised Kunitz proteins from other taxa: Simukunin (ACH56928.1) from the blackfly *Simulium vittatum*, EgKU8 (ACM79010.1) from *Echinococcus granulosus*, Fh-KTM (AAB46830.1) from *Fasciola hepatica,* Ixolaris (AAK83022.1) from the hard-tick *Ixodes scapularis*, BPTI (1510193A) and human TFPI-2 (AAA20094)

SmKI-1 is comprised of 146 amino acids and has a signal peptide of 20 residues. The mature protein has a putative molecular mass of 15.108 kDa and an isoelectric point of 8.22. One putative N-glycosylation site is predicted for the mature SmKI-1 protein. Phylogenetic analysis (Fig. 2) with SmKI-1 and other functionally characterised Kunitz proteins revealed a high relatedness of SmKI-1 with Simukunin, a Kunitz type plasma coagulation inhibitor from the salivary glands of the black fly *Simulium vittatum* [37]. BLASTP analysis revealed that SmKI-1 shared greatest sequence identity (63 % identity and 34 % query cover, E value 2x10^−21^) with Simukunin. SmKI-1 had the second highest sequence identity (57 % identity and 39 % query cover, E value 5x10^−18^) with human tissue factor pathway inhibitor-2 (TFPI-2) (GenBank No AAA20094.1), which is a three tandem domain Kunitz protein.

### *SmKI-1* gene expression

**Fig. 3:**
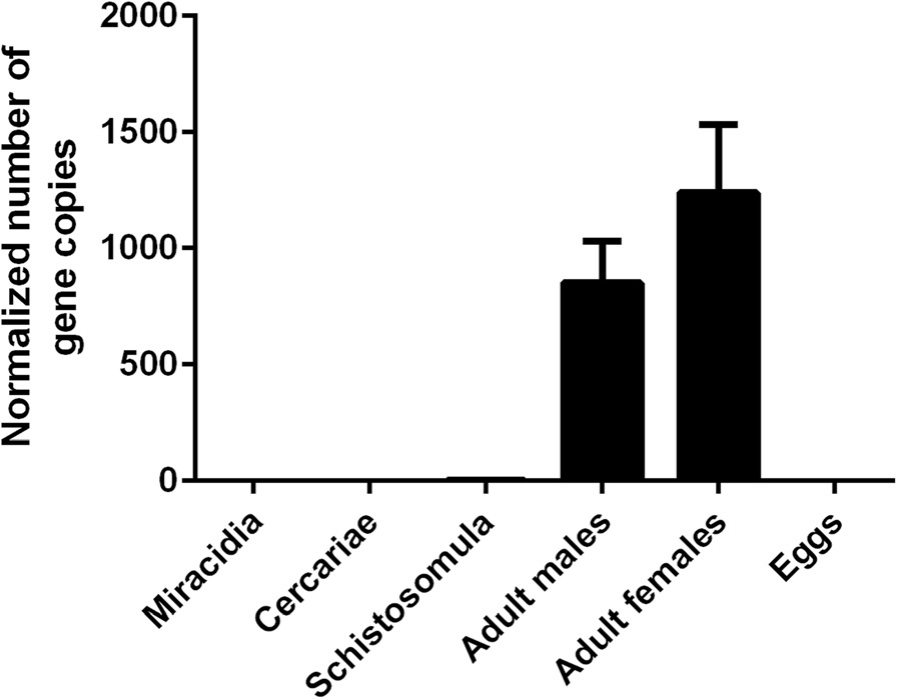
Real time PCR showing expression of the SmKI-1 gene after normalisation with DNA segregation ATPase as housekeeping gene. The error bars represent the mean ± standard error of the mean (SEM)

Real time PCR on preparations of cDNA from a range of *S. mansoni* life cycle stages indicated that the *SmKI-1* gene was highly expressed in both male and female adult worms but no expression was evident in miracidia, cercariae, schistosomula or eggs (Fig. 3).

### Protein purification and western blotting

**Fig. 4:**
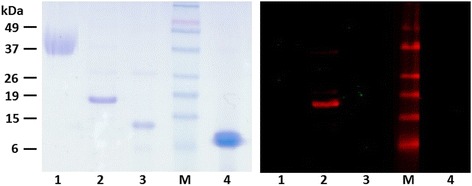
SDS-PAGE (left panel) (3 μg of each protein) and Western blotting (300 ng of each protein) with mouse anti-SmKI-1 polyclonal antibody (right panel): Lane 1, ulinastatin; lane 2, SmKI-1; lane 3, EgKI; lane 4, BPTI; M, Molecular weight markers (kDa)

A yield of 1.1 mg/ml recombinant SmKI-1 protein was obtained with ≥85 % purity from 800 ml total culture volume. The mouse anti-SmKI-1 polyclonal antibody was shown to be specific as no cross reactivity with other tested Kunitz proteins was evident (Fig. 4). Western blotting using this antibody to probe a whole soluble parasite antigen extract and ES products of adult *S. mansoni* revealed the presence of a low level of SmKI-1 but only in the ES products (not shown).

### Localisation of SmKI-1

**Fig. 5:**
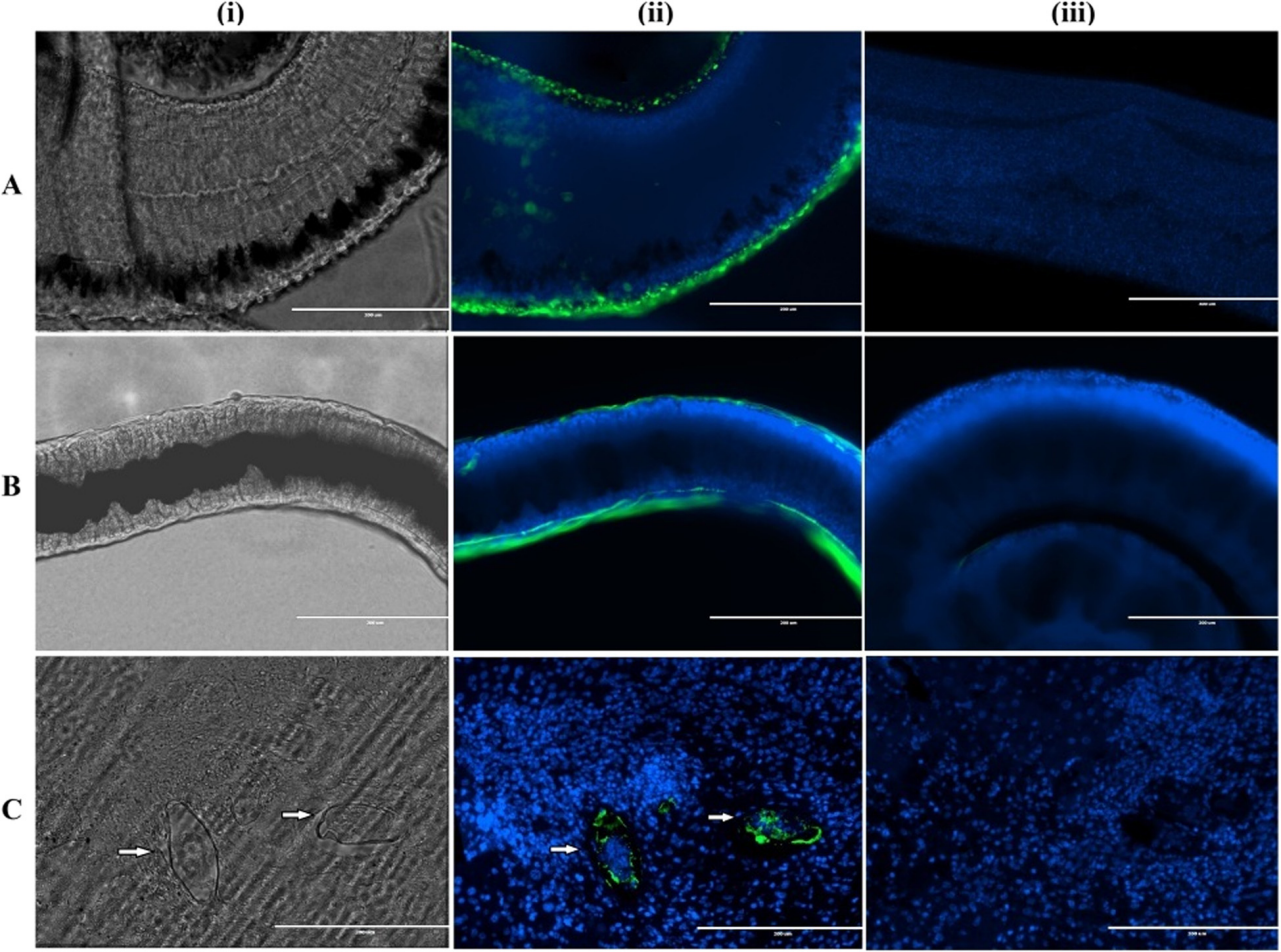
Immunolocalisation of SmKI-1 in adult male *S. mansoni* (**a**), adult female *S. mansoni* (**b**) and *S. mansoni* eggs (white arrows) trapped in infected liver tissue (**c**): (i). with white light; (ii). with anti-SmKI-1 antibodies and (iii) with negative control pre-immune mouse serum. Positive immunofluorescence for SmKI-1 is shown in green and the nuclei stained with DAPI appear blue. (Scale bar = 200 μm)

Immunolocalisation, using the mouse polyclonal anti-SmKI-1 anti-serum, indicated the presence of SmKI-1 along the tegument of adult worms, specifically in the tubercles of males and in the sub-shell region of eggs trapped in infected liver tissue (Fig. 5). No immunoreactivity was evident with cercariae, schistosomula or miracidia (data not shown).

### Coagulation assays

**Fig. 6:**
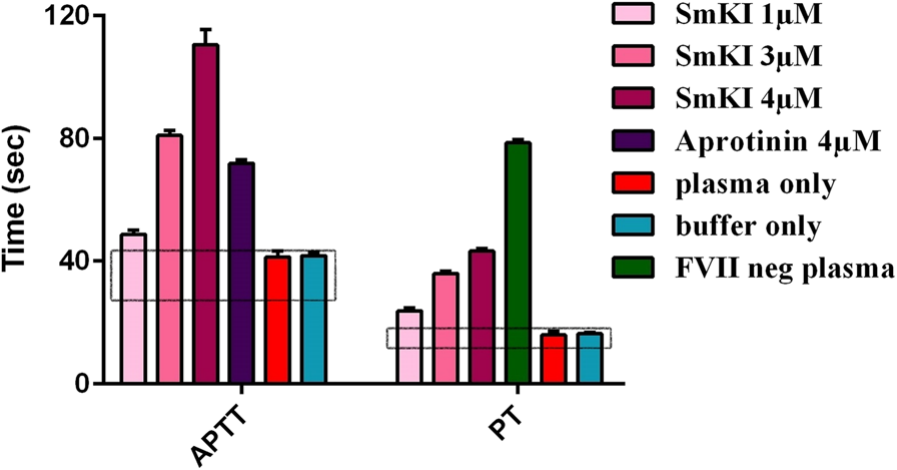
SmKI-1 prolongs both APTT and PT in clot formation. The normal time periods for clot formation during APTT and PT are shown by the rectangles. Aprotinin and FVII-deficient plasma were used as positive controls for APTT and PT, respectively. Error bars represent the mean ± SEM

**Fig. 7:**
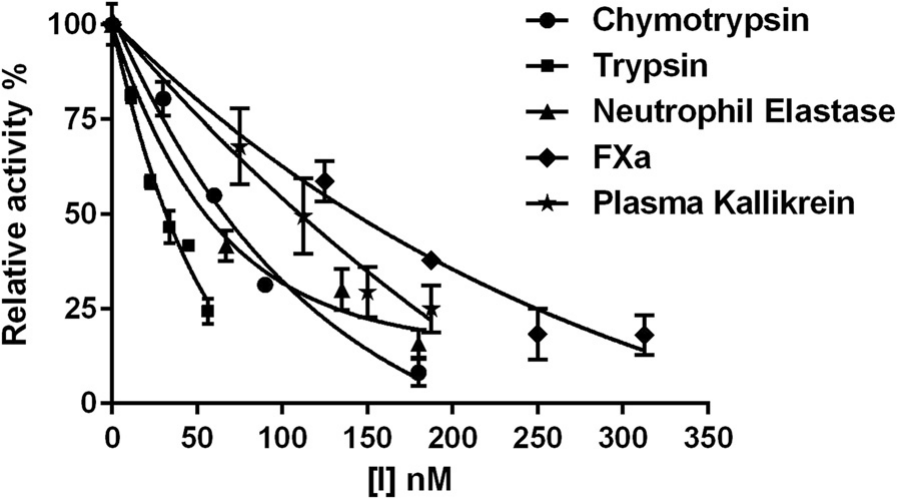
Percentage of relative inhibitory activity of rSmKI-1 against trypsin, chymotrypsin neutrophil elastase, FXa and plasma kallikrein. Error bars represent means ± SEM; [I], concentration of rSmKI-1

Recombinant SmKI-1 extended blood coagulation time in a dose dependent manner; i.e. in the presence of increasing rSmKI-1 concentrations clot formation was delayed in both the APTT and the PT (Fig. 6). The normal time period for clot formation in APTT is 26–41 s and in PT is 10–14 s. An approximately three-fold increase in the time taken for clot formation was observed in both assays when rSmKI-1 was present at a concentration of 4 μM. The prolongation in time of both APTT and PT suggests that SmKI-1 inhibits one or more of the coagulation factors involved in the intrinsic, extrinsic or common coagulation pathways. No direct thrombin inhibition was detected as clot formation was completed within the normal time period of 12–18 s for TCT (data not shown) which ruled out any interference of SmKI-1 with the conversion of fibrinogen to fibrin. Notably, SmKI-1 inhibited two tested coagulation associated serine proteases, FXa and plasma kallikrein, in the nanomolar range (Fig. 7).

### Protease inhibition assays

Purified rSmKI-1 inhibited trypsin, chymotrypsin, neutrophil elastase, FXa and plasma kallikrein with IC_50_ values of 35 nM, 61 nM, 56 nM, 142 nM and 112 nM respectively (Fig. 7), but had limited or no effect on cathepsin G or pancreatic elastase (data not shown). The results indicate that SmKI-1 is a potent trypsin inhibitor which is in agreement with the published literature that Kunitz proteins containing Arg at the P_1_ site are typical trypsin inhibitors. The inhibition of FXa and plasma kallikrein further supports the ability of SmKI-1 to inhibit blood coagulation and the inhibition of neutrophil elastase indicates its capability to interfere with inflammatory reactions.

## Discussion

Proteases play important roles in a variety of biological processes in higher animals, including immune defense, inflammation, blood clotting and tissue remodeling [38]. Serine proteases represent over one third of the known proteolytic enzymes and the Clan PA proteases, bearing the trypsin fold, are the largest family; most Clan PA proteins have trypsin-like substrate specificity and are involved mainly in blood coagulation and the immune response [39]. Neutrophils are the first line of defence in human innate immunity [40]. Recent findings suggest that neutrophil elastase, secreted by activated neutrophils, triggers inflammatory reactions as well as the initiation of blood coagulation by inducing the secretion of tissue factor [41] and inhibiting tissue factor pathway inhibitor (TFPI) [42]. Here we show that SmKI-1 inhibits several proteases, including neutrophil elastase, which is involved in triggering immune response reactions. Trypsin and chymotrypsin are serine protease digestive enzymes which degrade proteins in the small intestine. Secretion of SmKI-1 by schistosome eggs might indicate it has an additional function in providing protection from these proteolytic enzymes in the gut as many eggs traverse the intestinal wall and pass into the gut lumen prior to being excreted in human stool to the external environment.

The presence of the male and female worm pairs in the mesenteric veins of the small intestine host blood vessels leads to hypercoagulability through alterations in blood flow and endothelial function [43]. This feature and the negatively charged surface of schistosomes would be expected to trigger the activation of molecules involved in initiating blood coagulation. Worm pairs cause local anti-thrombogenic effects while the presence of eggs causes systemic effects [44]. However, the hypocoagulable and hyperfibrinolytic state of hepatosplenic schistosomiasis patients indicates that schistosomes are capable of suppressing the host haemostatic response and, preventing thrombus formation [43]. Factor XII in blood is activated when exposed to a negatively charged surface, such as that presented by *S. mansoni*, which then triggers the intrinsic coagulation pathway. A cascade of reactions subsequently leads to the activation of the common coagulation pathway which ultimately results in the formation of a fibrin clot [44]. We show here that SmKI-1 can inhibit the proteolytic activities of coagulation factor Xa, also known as prothrombinase, thrombokinase or thromboplastin, and plasma kallikrein, another serine protease involved in coagulation that generates plasmin from plasminogen and liberates kinins from kininogens. The inhibition of FXa and plasma kallikrein in the nanomolar range and prolongation of the APTT and PT by SmKI-1 suggests this Kunitz protein likely plays a role in interfering with the mammalian host coagulation pathways. Even though a relatively high concentration of SmKI-1 (micro-molar range) was needed for a visible positive reaction in the coagulation assays we performed, the observed inhibition of PK and FXa in the nanomolar range suggests that a lower concentration of SmKI-1 would result in an effective level of anti-coagulant activity *in vivo*.

It has long been documented that the excretory-secretory (ES) products of the parasitic worms can modulate the expression of host immune responses [45]. Schistosome ES products, which act at the interface between these blood flukes and the mammalian host, may be responsible for the capacity of schistosomes to redirect the host immune system, thereby modulating the immune response [46]. Recent advances in genomics, transcriptomics and proteomics have shown that these ES products contain soluble mediators which ligate, degrade or interact with host immune cells [47, 48]. Helminth ES products predominantly consist of proteases, protease inhibitors, venom allergen homologues, glycolytic enzymes and lectins [46]. Even though the qPCR showed high mRNA expression of SmKI-1, low protein expression was evident in adult ES products. Several possible reasons have been discussed previously for the poor correlation between the levels of mRNA and protein expression, the main one being the complicated and variable post-translational mechanisms involved [49, 50]. SmKI-1 may be secreted as a response to an external stimulus such as the presence of a protease. With *in vitro* experiments it is difficult to determine precise mechanisms taking place *in vivo*. It is noteworthy, however, that SmKI-1 has not been identified in recent reports of the *S. mansoni* proteome [51–53]. Proteomic studies generally identify the most abundant ES proteins but they may miss those that are bioactive but which are present at a low level [46].

The outer surface of the adult schistosome tegument is a unique double membrane structure which plays a crucial role in modulating host responses and ensuring parasite survival [54]. As a tegumentally localised protein, released in the ES, we propose that SmKI-1 is intimately involved in providing protection to the parasite in its mammalian host. Resident in mesenteric veins, schistosomes release eggs into the blood circulation where they are also exposed to host attack. This may stimulate the eggs to produce and secrete biologically important proteins such as SmKI-1, which can provide protection from the immune system. In support of this, we have shown by immunolocalisation that SmKI-1 is present between the outer shell and the developing miracidia in eggs trapped in the infected mouse liver. However, there was no immunoreactivity of polyclonal anti-SmKI-1 antibodies with purified, isolated eggs or soluble egg antigens (SEA) obtained from the purified eggs of *S. mansoni*. Neither were we able to detect *SmKI-1* gene expression in the purified eggs by real time PCR. A possible reason could be that mRNAs of some genes are less stable than proteins [55] and the extensive, time consuming collagenase-based procedure we used to purify *S. mansoni* eggs from infected mouse livers likely caused the inhibition of *SmKI-1* mRNA expression. In addition, the SmKI-1 protein could have been washed away during the purification procedure. In contrast, fixing infected liver tissue soon after it was obtained, likely preserved the integrity of SmKI-1 and its expression in eggs trapped in the liver tissue.

The Smp_147730 gene, encoding the Kunitz inhibitor characterised here, was previously identified following transcriptome profiling of mechanically transformed, but not skin transformed, *S. mansoni* schistosomula [21]. Depending on which cercarial transformation method is used, RNA transcription and protein expression can vary [56]. The method we used in this study to transform schistosomula was different from the earlier method used [21] which might explain why we did not observe upregulation of the *SmKI-1* gene in schistosomula. In this context, it is noteworthy that significant differences in gene expression profiles have also been reported between mechanically transformed and *in vivo* obtained lung schistosomula of *S. japonicum* have also been reported [57]. It is likely that schistosomula transiently express and secrete key proteins, including SmKI-1, soon after skin penetration, in order to survive host immune attack.

Schistosomes exhibit remarkably diverse mechanisms to regulate their interactions with the mammalian host and often use molecular mimicry [58]. TFPI is a naturally occurring coagulation inhibitor which regulates the human coagulation pathway. It is also a Kunitz type protein containing three domains with dual inhibitory actions whereby it binds to the tissue factor/FVIIa complex to prevent it from acting on FIX and FX substrates as well as directly inhibiting FXa [59]. Having high sequence similarity to TFPI, SmKI-1 may have evolved to mimic the functions of this human Kunitz protein.

## Conclusion

Our characterisation of SmKI-1 suggests that this Kunitz protein likely plays a specific and essential biological role in protecting the fluke from the onslaught of the host defenses through the inhibition of serine proteases involved in coagulation and inflammation, and may therefore be a good candidate for future evaluation as a vaccine or drug target. Moreover, its inhibition of FXa and plasma kallikrein and the prolongation of blood clotting time point towards the potential clinical application of SmKI-1 in anti-thrombotic therapy. Further studies are needed to characterise the other putative Kunitz inhibitors we have identified in order to further increase our knowledge of schistosome defense mechanisms.

## Additional file

Additional file 1:
**Putative** ***S. mansoni*** **Kunitz proteins.** The Kunitz domains are highlighted in grey, conserved cysteine residues in blue, non-matching features in red, P1 reactive site in black, chitin binding type-2 domains in purple and spondin domain in green. (PDF 75 kb)
