# Supplementary material for: Functional expression of a novel Kunitz type protease inhibitor from the human blood fluke Schistosoma mansoni
Source: Parasit Vectors. 2015 Aug 4;8:408. doi: 10.1186/s13071-015-1022-z (PMC4524284; doi:10.1186/s13071-015-1022-z)
Supplement: Additional file 1: — Putative S. mansoni Kunitz proteins. The Kunitz domains are highlighted in grey, conserved cysteine residues in blue, non-matching features in red, P1 reactive site in black, chitin binding type-2 domains in purple and spondin domain in green. (PDF 75 kb) [file 13071_2015_1022_MOESM1_ESM.pdf]

Additional File 1

Additional File 1: Putative *S. mansoni* Kunitz proteins. The Kunitz domains are highlighted in grey, conserved cysteine residues in blue, non-matching features in red, P_1 reactive site in black, chitin binding type-2 domains in purple and spondin domain in green.

>Smp_139840
MAQEKESLVESHWSDFQPPPSSGIIYKREVIPRHRTLSALLIGLGIIVTLILLLVLSYFMHVYSVVHS
HINMQDTLNPICLLPRLTGKCRASFNRWGWNPQTTTCEEFIYGGCDANENNFLTKEECETVFELLMAT
LNTGLLTETPE

>Smp_147730
MFSFCLYGTLLLICVQSVASYRKGNSDCLLDYDEGICRALLKRFYYDSVNQTCEIFYYGGCLGNGNNF
LSKEECERKCGGQIYMTEKSFETTKQMETTSTSIDRSDNTETTITTQKPLSVGAKIVLGILDIKNKVS
NLFKKIKGEK

>Smp_147710
MEVKLLIVLVLFVVCHLVLCASLREQCLQPPQIGKGDSNLTRFYYDKSKNQCIQFFYKGRNSDRNNFR
TKIACEWYCVVKKRK

>Smp_179120
MKKGYCLQNKPRFYYNPAENKCLPFSFSGCGGNENRFHTMEKCESFCKKTIVGTENKKPSTTPAGQRT
NEPTTRIIYITKSSRRK

>Smp_180810
MTASVKNDDDNDYVDTSDSSEVTNTLEYEEREDGVGEQDNVTEEIAFNSKESYNSLADEQAQLESLNR
NQFEIETNDSDDNTEIITALHIKSSNFENEDGIGITESTESQPLLNTITEETLSLVTTDNNLGVLSLN
TDDHIPSSHSTDTDESLYSDYILEPNDIVEIPSQSQEHVNVTEEILGNKNEIILDKLEELEGTDQKLD
QTTIVPEVLTNEMKVSLNTDKPSSELSKQDIQVTEILADTISLSTTLQSDSNTSAHEKTSIIKLVSIS
TNRQPVERTYERNVLIEKCYQYPKIKICNKLTDMDLKHQGGWYYNPTEMQCQYTSDCIINDNYFKTKN
ECIETCEYWRGTARCLAPPQAGHFRCSAVNTEKTIRPILMVYFDKVSGKCRWFTYYGCGGSANRFTSI
TECENTCGNLIAYKILLVASQLCQVAPTTQLFPQWSRIEMDTWSEISYDGQFYSKEKSEPILFDNLTY
IDAMRIEPECANVGQHESRWYLDVETNQCQEFAYSHCGGSSNNFLTRADCEQFCGATKPDISAICKLK
PSFGECTGYKTMWYYDPNWVMNMDGQDHYLIGGCKQFNYSGCGGNSNRFPTQAACELTCQFNTKVELQ
INIDPVEGLNNVTTRSEPIRNKDNNQSTIVITESNKTIHNNLVKKMTKLTLMNIDLEPCRRHPVFGFC
RPLNCSEEHRRNQTCHFLNFQRWFFNAHTSNCEAYSYSGCGASENTFDDAQACQAACKARIVRPDRDQ
RCDSNPHTKKCYTLSVSGSNQQDVSSEKDIIAFHFSMSSATCKAFHLNTKRPGCSMEQYFPNGYDCLR
ECVKSSPNEKHLQNRCFARKTHVLWNCTDQSVKTYRWSYLPEINQCVRFSECTNPDQIIEQPGNNFAS
RSECETTCMASSFEEVCQLPKDPGPCTSFQTRYFYDSSTSKCRVFLYGGCLGNFNRFLLRKECELACS
QFSTHILSTSNHTKPQENSMEETLVGYDESPPLSAQVFEKMKQITQHHTDRYPWDICLDSHSYGTCSL
SGGQYQTTSEYPYVPLTRYYYDRRLGQCQPYTYTGCGARGNHFDTLEKCQIVCENRLKNPKFARCHYD
KPITKCPGSGIQAWAYNHTIGDCYFFEICEPEEKESEFQIPQKKFSFRLRRTSQWLGVWQARTGNLPE
GIYATRSACQYHCLPKPPRGTDTQNVCHMNPIVTVPFGCNAMVTRWYFEPRETQCRSYITCPQYGNNF
PSHKACQDICTPGHPIDVCRLPYDHGGCSNFEKRWYYDMQKRMCMPFTYGGCFGNSNRFVTKAECEGF
CMGKDKNSTDPSYPERFYYDHSRKQCLPFRFTGLLAHGNNFPSLSACNATCIYVPDIEVIAEKIMQSS
NLNSDKKLEESNKTLIQASSMENPTNTQQMTDENTSISVHRIPTDNKNILSEKFPCLPFITSSQYENI
DRHTFCNSEDIIHELGYRFQVTSSVHVTDETIDGICVPILVPICLNEPQRLYGQLNTYESQMIGRVFK
TEAECEETCLLKNRFKRSIIMEKSFDTTKNIRQLLNKILDEKSKLSGHLNDSVYPTLNYEMITDKQHN
KIFQAVTHSFNQSNFKKTLTLEVLENEHIKLPCWIVSQNKPHVQWIHLLTGKRYPVTVHHHSKNKNIW
ISHLLVHNTKTQLHTGGWVCEASDLETTEYAKATIILNVVSLQPNKILDMSSQNTVSSHDTIMVPKDG
LVFLSCPHYQYLGDTLWKKNGEDILQANSEYLVIEHANPKFHTGIWICGINYDNNFIELYKFNISVGY
PPELQKSNTNLSKAISQFPLTCPINTQGEPAGYVQWFECKHTNNCYPLTTELYSSMLNQIILIMCRIE
NIWGMDETYFNLTTE

>Smp_052230

MKISQVTLIMNILHLLFIQSTESYAVLEGKQIVKKVYTKRQPIVLTAAAGFMSSTNSIETMNNSNANR
SIFNNGNGFSTIHHVPKNSTGEYIPPNFITGAHLAPAHSIFSDINKENYTKWSFSTTFNPSTPSQLNK
EVNEDILTDFPLHEQQNQTEGETHSFGGGNQQQIMETDNDFPQIHSDYLYDANYDFPEEMNKYQVVIS
SDMDPPYSMDQPNGAAINSSKFLWSYNRDYDQPMEENVQFKQNIYVDNQQKKEEARPVYHSPVRWAIR
QIATNGQPRHIACSQPLDRGVGSNELSSWYYDSNDGRCRWFGYRGYGGNANRFYSRTACEELCVRDNQ
NLCEFAKCPKSITTCELVGDQSCKVYKQQHSKSWEAECPPDQPVCVTKRNTRMAPDIEFENVPSECKQ
PPDAGSCQIKNPSQNFFYDIESNDCTSFYFHECGGNDNRFVTKSECMSHCSP

>Smp_180240

MLSKCYQMCLACNIGYFKLSVIFHFLLFSFITFTNFDPILCLNRYNKVPNDCSEMPEYSSVIRRPGDG
GLEIRIQLLPPDAKRRRGSGNNGHVILHSSDLISPNYKPAVHYLNGRDYLITVGPKLHSDTAKNGLRL
ESVYLTAVPLGSPESEEFHQSKGRFMPTVCSNHAIGAIRFAYNFPNHVSFVWTSPANSESTTLKYTPN
AQSFNSCLEIRATIIPFHWHRFYFKNSGSLRHVLCPAEDLRQIDEWSEYLHSNTKLFRKVKSDNVKAR
SITNIGKFDNDDSKNHRYSDSGYYGTEELDSCSVQELPKPVRTCCACNTAIYRLIIQSEWQQQHWRD
WPTTLTGAGASHSPQYDIFHAGGYASPAVDALCTTSDVSKLESEFRNQAGENILTVIRTRGIDSMAPP
EQRFRSALFAVNSSHHLLSFLSRIVPSPDWCTGLSRVDICLQNCSWPLRMQFRLEPWDAGVMTGNTYI
PIETSERLREPKPMCPITPDLRPNTPFTVLTDYVSLPNSDSFSLTNLHTSGSNTFRSPNLGLPMSDQM
NLLNIPSTSGRMRRQFARLGTVELELLRINEHESCATEVLQEDQAMSKASNFPRRDSYGGSFDTRTGT
TESDAKRAPADLLGARCHLSEWSPWSPCTQVDLDTCSTSEAKSFWTNTPPRMWRTRHSISQSNDEDCL
AAKLKEEKACEMPTAKQTCGPIPISTPHTRMNFMDTCNKLPWGPWSTCINATCTRPGMIYRWRRFPDQ
ASKVACERHPLASQVDTCWPPSNFQCSLSELQTACLEEPPTPQRLCVRPLNTTKRYYYSSATKQCKEF
EYVPECHLRALVANHPISLTRNVFKDRNSCEQLCTNKQIQADAVWNPIVKRETEKLQHRCEQSLMKGS
LCQSNKRSYSWYYDKQMNRCISFEYLGCYGNENNFRSERECIT
